# Fast T2 gradient-spin-echo (T2-GraSE) mapping for myocardial edema quantification: first in vivo validation in a porcine model of ischemia/reperfusion

**DOI:** 10.1186/s12968-015-0199-9

**Published:** 2015-11-04

**Authors:** Rodrigo Fernández-Jiménez, Javier Sánchez-González, Jaume Aguero, María del Trigo, Carlos Galán-Arriola, Valentin Fuster, Borja Ibáñez

**Affiliations:** Centro Nacional de Investigaciones Cardiovasculares Carlos III (CNIC), Madrid, Spain; Hospital Universitario Clínico San Carlos, Madrid, Spain; Philips Healthcare, Madrid, Spain; The Zena and Michael A. Wiener CVI, Mount Sinai School of Medicine, New York, NY USA; Department of Cardiology, Instituto de Investigación Sanitaria, Fundación Jiménez Díaz Hospital, Madrid, Spain

**Keywords:** Cardiovascular magnetic resonance, T2-mapping, Imaging, Myocardial infarction, Edema, Water content

## Abstract

**Background:**

Several T2-mapping sequences have been recently proposed to quantify myocardial edema by providing T2 relaxation time values. However, no T2-mapping sequence has ever been validated against actual myocardial water content for edema detection. In addition, these T2-mapping sequences are either time-consuming or require specialized software for data acquisition and/or post-processing, factors impeding their routine clinical use. Our objective was to obtain in vivo validation of a sequence for fast and accurate myocardial T2-mapping (T2 gradient-spin-echo [GraSE]) that can be easily integrated in routine protocols.

**Methods:**

The study population comprised 25 pigs. Closed-chest 40 min ischemia/reperfusion was performed in 20 pigs. Pigs were sacrificed at 120 min (*n* = 5), 24 h (*n* = 5), 4 days (*n* = 5) and 7 days (*n* = 5) after reperfusion, and heart tissue extracted for quantification of myocardial water content. For the evaluation of T2 relaxation time, cardiovascular magnetic resonance (CMR) scans, including T2 turbo-spin-echo (T2-TSE, reference standard) mapping and T2-GraSE mapping, were performed at baseline and at every follow-up until sacrifice. Five additional pigs were sacrificed after baseline CMR study and served as controls.

**Results:**

Acquisition of T2-GraSE mapping was significantly (3-fold) faster than conventional T2-TSE mapping. Myocardial T2 relaxation measurements performed by T2-TSE and T2-GraSE mapping demonstrated an almost perfect correlation (R^2^ = 0.99) and agreement with no systematic error between techniques. The two T2-mapping sequences showed similarly good correlations with myocardial water content: R^2^ = 0.75 and R^2^ = 0.73 for T2-TSE and T2-GraSE mapping, respectively.

**Conclusions:**

We present the first in vivo validation of T2-mapping to assess myocardial edema. Given its shorter acquisition time and no requirement for specific software for data acquisition or post-processing, fast T2-GraSE mapping of the myocardium offers an attractive alternative to current CMR sequences for T2 quantification.

## Background

Cardiovascular magnetic resonance (CMR) has emerged as a popular and useful tool for noninvasive myocardial tissue characterization [1]. CMR provides valuable anatomical and functional information through high spatial resolution images and soft tissue contrast, without exposing patients to ionizing radiation. There is particular interest in using CMR to detect and track myocardial water content, because edema is a feature of many cardiovascular conditions [2–4]. T2-weighted (T2W) CMR sequences have been used for this task [5], but several problems inherent to these sequences have limited the widespread acceptance of this sequence to detect edema [6]. New T2-mapping sequences have recently been proposed to overcome some of these limitations [7–10] and provide absolute quantification of myocardial T2 relaxation times that can be compared among studies, the reference standard being T2 turbo-spin-echo (T2-TSE) [11, 12]. However, these methods are either time-consuming or require specialized software for data acquisition and/or post-processing, factors which limit their routine clinical use.

It is noteworthy that no T2-mapping sequence has ever been validated for quantification of myocardial water content against direct measurement by a gold standard technique. The validity of T2-mapping sequences for this task has been assumed based on their ability to retrospectively delineate the hypoperfused myocardial territory supplied by the occluded coronary artery (the area at risk). However, regional T2 relaxation time in the post-ischemia/reperfusion area is affected by tissue characteristics [13, 14], the application of cardioprotective therapies [15, 16], and the timing of image acquisition [17], and therefore the evidence supporting the validity of T2-mapping for myocardial edema quantification is weak.

In this study, we sought to provide in vivo validation of a sequence for fast and accurate T2-mapping of the myocardium using the gradient-spin-echo (GraSE) technique [18], which could be rapidly and easily integrated in daily protocols as it is commercially available from many vendors. To achieve this goal, we used a closed-chest pig model of ischemia/reperfusion in which animals were serially scanned and sacrificed at different time-points after reperfusion for direct quantification of myocardial water content.

## Methods

### General considerations and study design

**Fig. 1:**
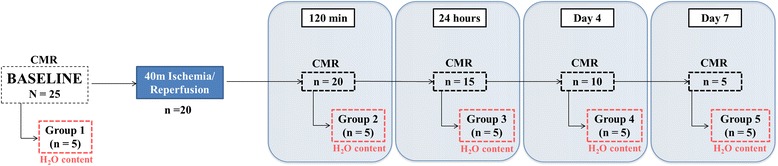
Study design. The study population comprised 5 groups of pigs (*n* = 5/group). Groups 2 to 5 were subjected to 40 min ischemia/reperfusion (I/R) and were sacrificed at different time-points during the first week after reperfusion and heart tissue extracted for direct quantification of myocardial water content. Five pigs (Group 1) were sacrificed with no intervention other than baseline cardiovascular magnetic resonance (CMR), and served as controls. CMR scans including T2-TSE and T2-GraSE mapping sequences were performed at all follow-up stages until sacrifice, so that animals sacrificed at day 7 underwent baseline, 120 min, 24 h, day 4 and day 7 CMR

Experimental procedures were performed in castrated male Large-White pigs weighing 30 to 40 kg. The study population comprised a total of 25 pigs. The experimental protocol was approved by the Institutional Animal Research Committee and conducted in accordance with recommendations of the Guide for the Care and Use of Laboratory Animals. The study design is summarized in Fig. 1. Briefly, reperfused acute myocardial infarction was induced in 20 pigs by closed-chest 40-min left anterior descending coronary artery occlusion followed by reperfusion. Pigs were sacrificed at 120 min (*n* = 5, Group 2), 24 h (*n* = 5, Group 3), 4 days (*n* = 5, Group 4) and 7 days (*n* = 5, Group 5) after reperfusion. CMR scans, including T2-TSE mapping (current standard) and T2-GraSE mapping sequences were performed at every follow-up until sacrifice (animals sacrificed at day 7 thus underwent baseline, 120 min, 24 h, day 4, and day 7 CMR). Five pigs (Group 1) were sacrificed with no other intervention than baseline CMR, and served as controls (healthy non-infarcted heart). Animals were sacrificed immediately after the last follow-up CMR, and myocardial tissue samples from ischemic and remote areas were rapidly collected for determination of water content.

### Myocardial infarction procedure

The protocol of ischemia/reperfusion has been detailed elsewhere [17]. In summary, anesthesia was induced by intramuscular injection of ketamine (20 mg/kg), xylazine (2 mg/kg), and midazolam (0.5 mg/kg) and maintained by continuous intravenous infusion of ketamine (2 mg/kg/h), xylazine (0.2 mg/kg/h) and midazolam (0.2 mg/kg/h). All animals were intubated and mechanically ventilated with oxygen (inspired O_2_ 28 %). Central venous and arterial lines were placed and a single bolus of unfractioned heparin (300 mg/kg) was administered before any further procedure. The left anterior descending coronary artery immediately distal to the origin of the first diagonal branch was occluded for 40 min with an angioplasty balloon introduced thorough a catheter inserted via the percutaneous femoral route. Balloon location and state of inflation were monitored angiographically. After balloon deflation, a coronary angiogram was recorded to confirm patency of the coronary artery. A continuous infusion of amiodarone (300 mg/h) was maintained during the procedure in all pigs to prevent malignant ventricular arrhythmias. In cases of ventricular fibrillation, a biphasic defibrillator was used to deliver non-synchronized shocks. At intermediate follow-up time points, animals were recovered and cared for by a dedicated team of veterinarians and technicians.

### CMR protocol

**Fig. 2:**
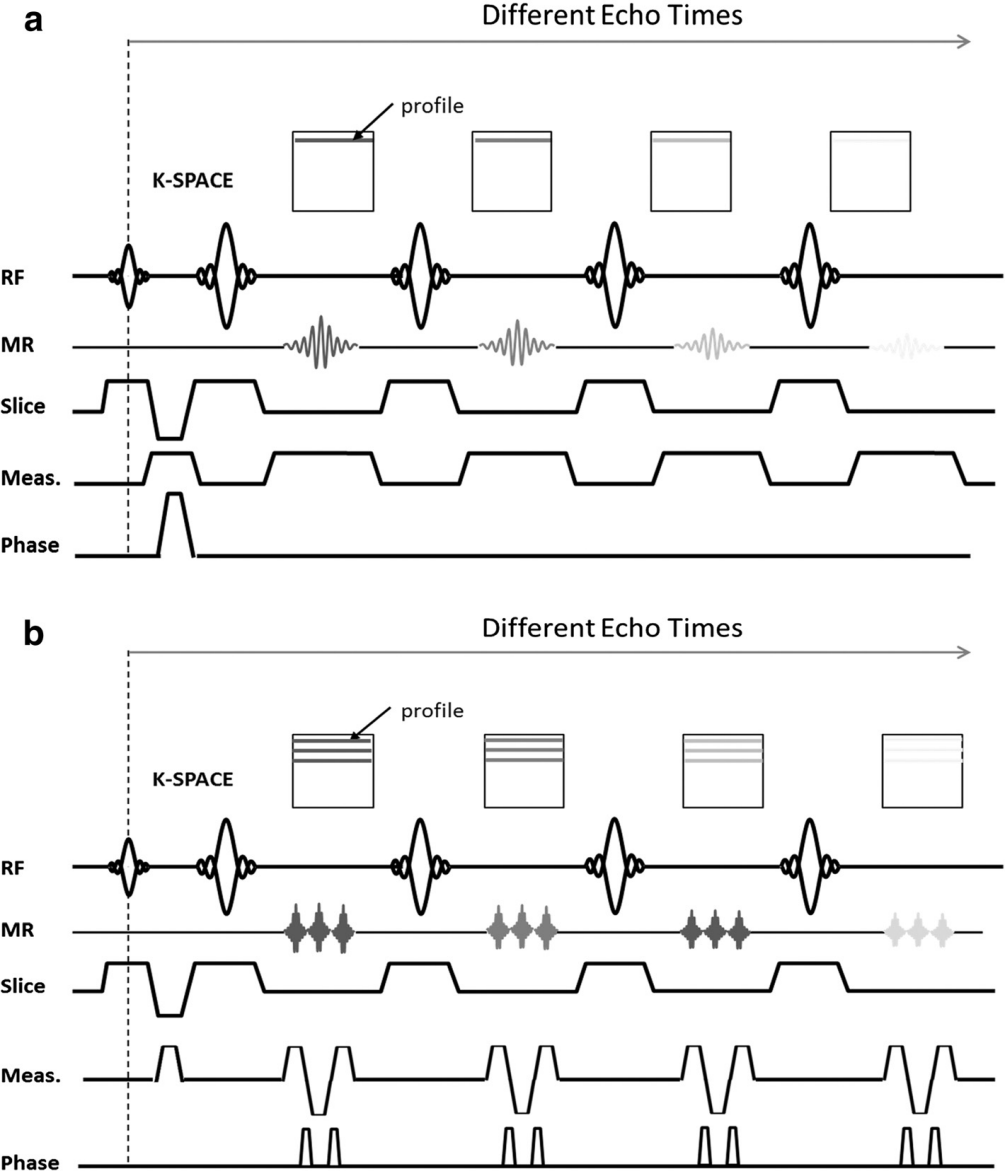
Detail of T2-TSE and T2-GraSE mapping sequences. General scheme of Turbo Spin Echo (TSE) and Gradient Spin Echo (GraSE) mapping sequences. For the TSE a single k-space line is acquired for every excitation requiring as many excitations as k-space lines in the image (**a**). Conversely, for the GraSE sequence an echo planar imaging (EPI) readout is interleaved between each refocusing pulse, so that as many k-space lines are acquired as there are EPI factors, thus allowing shorter scan times (**b**). RF: radiofrequency pulse. MR: magnetic resonance signal. Meas.: measurement encoding

Baseline CMR was performed immediately before myocardial infarction and subsequently repeated at post-infarction follow-up time points until sacrifice. All scans were performed during free breathing in a Philips 3-T Achieva Tx whole body scanner (Philips Healthcare, Best, the Netherlands) equipped with a 32-element phased-array cardiac coil. The imaging protocol included a standard segmented cine steady-state free-precession (SSFP) sequence to provide high quality anatomical references, a T2- turbo spin multi-echo mapping sequence (T2-TSE), and a T2- gradient spin echo mapping sequence (T2-GraSE). The imaging parameters for the SSFP sequence were FOV of 280x280, slice thickness of 6 mm with no gap, TR 2.8 ms, TE 1.4 ms, flip angle 45°, cardiac phases 30, voxel size 1.8×1.8 mm^2^, and 3 NEX. The imaging parameters for the T2-TSE mapping were FOV 300×300 with and acquisition voxel size of 1.8×1.8 mm^2^ and slice thickness 8 mm, TR 2 heartbeats, and ten echo times ranging from 4.9 to 49 ms. The imaging parameters for the T2-GraSE mapping were FOV 300×300 with an acquisition voxel size of 1.8×2.0 mm^2^ and slice thickness 8 mm, TR 2 heartbeats, and eight echo times ranging from 6.7 to 53.6 ms, EPI factor 3. Both T2 mapping sequence where black blood triggered with a trigger delay placed at mid-diastole. Both T2-mapping sequences are schematized in Fig. 2. SSFP was performed to acquire 13–15 contiguous short axis slices covering the heart from the base to the apex, whereas T2-maps were acquired in a mid-apical ventricular short axis slice corresponding to the same anatomical level in all studies, in order to track T2 relaxation time changes across time.

### CMR data analysis

CMR images were analyzed using dedicated software (MR Extended Work Space 2.6, Philips Healthcare, The Netherlands). T2-maps were automatically generated on the acquisition scanner by fitting the SI of all echo times to a monoexponential decay curve at each pixel with a maximum likelihood expectation maximization algorithm. T2 relaxation maps were quantitatively analyzed by placing a wide transmural region of interest (ROI) at the ischemic and remote areas of the corresponding slice in all studies. The masking was defined in the first echo image to improve the contrast between the cardiac muscle and the cavity. Higher T2 values in this interface can be found due to slow flow artifact; therefore, ROIs were carefully placed avoiding those areas from the analysis to minimize contamination on the reported T2 values. Hypointense areas suggestive of microvascular obstruction or hemorrhage were included in the ROI for T2 quantification purposes.

### Quantification of myocardial water content

Paired myocardial samples were collected within the first 5 min of sacrifice from the infarcted and remote myocardium of all pigs. Tissue samples were immediately blotted to remove surface moisture and introduced into glass containers previously weighed on a high-precision scale. The containers were weighed before and after drying for 48 h at 100 °C in a desiccating oven. Tissue water content was calculated as follows: water content (%) = [(wet weight − dry weight)/wet weight] × 100. An empty container was weighed before and after desiccation as an additional calibration control.

### Statistical analysis

Normal distribution was checked using graphical methods and a Shapiro-Wilk test. For quantitative variables showing a normal distribution, data are expressed as mean ± standard deviation. For quantitative variables showing a non-normal distribution, data are reported as medians with first and third quartiles. Agreement of T2 relaxation time measurements between T2-TSE and T2-GraSE mapping techniques was evaluated by the square of Pearson’s correlation coefficient, the intraclass correlation coefficient for two-way random effect models, and Bland-Altman analysis [19]. Association between T2 relaxation time measurements performed by T2-TSE and T2-GraSE, and water content was evaluated by the square of Pearson’s correlation coefficient. The significance of the difference between these correlation coefficients was performed using the Fisher r-to-z transformation and the *cortesti* user-written command for Stata. Statistical significance was set at a two-tailed probability level of 0.05. All statistical analyses were performed using commercially available software (Stata 12.0). The authors had full access to the data and take responsibility for its integrity.

## Results

### Duration of sequence acquisition and T2 relaxation time measurements performed by T2-TSE and T2-GraSE mapping

Mean study acquisition length was 189 ± 19 s for T2-TSE mapping and 65 ± 8 s for T2-GraSE mapping (*p* < 0.001). T2 relaxation time measurements obtained by T2-TSE and T2-GraSE mapping at different follow-up points in the ischemic and remote myocardium are summarized in Table 1 and Table 2, respectively.

**Table 1:** T2 relaxation time (ms) in the ischemic myocardium measured by T2-TSE and T2-GraSE mapping sequences at different time-points during the first week after ischemia/reperfusion

T2 relaxation time (ms)						
		Baseline	R-120 min	R-24 h	R-Day4	R-Day7
Group 1 (Control)	TSE	47.7 (4.0)				
	GraSE	48.0 (4.7)				
Group 2 (I/R-120 min)	TSE	48.7 (0.6)	73.3 (10.0)			
	GraSE	49.9 (2.5)	76.5 (7.8)			
Group 3 (I/R-24 h)	TSE	46.5 (1.9)	72.4 (12.3)	45.9 (5.3)		
	GraSE	48.3 (4.8)	73.9 (10.0)	42.9 (4.5)		
Group 4 (I/R-4 days)	TSE	45.9 (1.6)	73.5 (4.2)	42.7 (9.3)	55.1 (13.2)	
	GraSE	45.0 (3.7)	78.7 (10.8)	42.6 (8.5)	54.3 (14.1)	
Group 5 (I/R-7 days)	TSE	47.2 (3.5)	72.6 (14.2)	47.0 (2.9)	64.9 (7.9)	78.4 (10.6)
	GraSE	46.5 (3.0)	74.8 (14.4)	46.7 (4.9)	66.4 (8.3)	78.9 (11.7)
Pooled	TSE	47.2 (2.6)	72.9 (9.9)	45.2 (6.2)	60.0 (11.5)	78.4 (10.6)
	GraSE	47.6 (3.9)	76.0 (10.3)	44.1 (6.1)	60.4 (12.6)	78.9 (11.7)

Values are mean (standard deviation). I/R: ischemia/reperfusion. TSE: turbo spin echo. GraSE: gradient spin echo. CMR T2 relaxation times measured by T2-TSE mapping in the ischemic myocardium of these pigs have been reported [17]

**Table 2:** T2 relaxation time (ms) in the remote myocardium measured by T2-TSE and T2-GraSE mapping sequences at different time-points during the first week after ischemia/reperfusion

T2 relaxation time (ms)						
		Baseline	R-120 min	R-24 h	R-Day4	R-Day7
Group 1 (Control)	TSE	46.1 (1.5)				
	GraSE	45.7 (1.8)				
Group 2 (I/R-120 min)	TSE	46.8 (1.8)	47.0 (1.0)			
	GraSE	47.2 (1.6)	48.4 (2.0)			
Group 3 (I/R-24 h)	TSE	46.2 (2.6)	48.6 (3.0)	45.2 (0.6)		
	GraSE	46.8 (1.9)	47.2 (5.2)	44.8 (1.2)		
Group 4 (I/R-4 days)	TSE	45.5 (0.8)	48.3 (4.0)	47.5 (3.1)	48.2 (2.9)	
	GraSE	44.8 (1.1)	45.5 (4.6)	45.9 (3.9)	49.1 (1.4)	
Group 5 (I/R-7 days)	TSE	46.7 (1.5)	48.5 (3.7)	51.4 (5.0)	50.1 (1.8)	50.0 (3.3)
	GraSE	44.6 (2.3)	46.5 (2.9)	51.0 (3.5)	49.0 (1.5)	49.3 (5.0)
Pooled	TSE	46.3 (1.7)	48.1 (3.0)	48.0 (4.1)	49.1 (2.5)	50.0 (3.3)
	GraSE	45.8 (1.9)	47.0 (3.7)	47.2 (4.0)	49.0 (1.4)	49.3 (5.0)

Values are mean (standard deviation). I/R: ischemia/reperfusion. TSE: turbo spin echo. GraSE: gradient spin echo. CMR T2 relaxation times measured by T2-TSE mapping in the remote myocardium of these pigs have been reported [17]

### Agreement of T2 relaxation time measurements performed by T2-TSE and T2-GraSE mapping

**Fig. 3:**
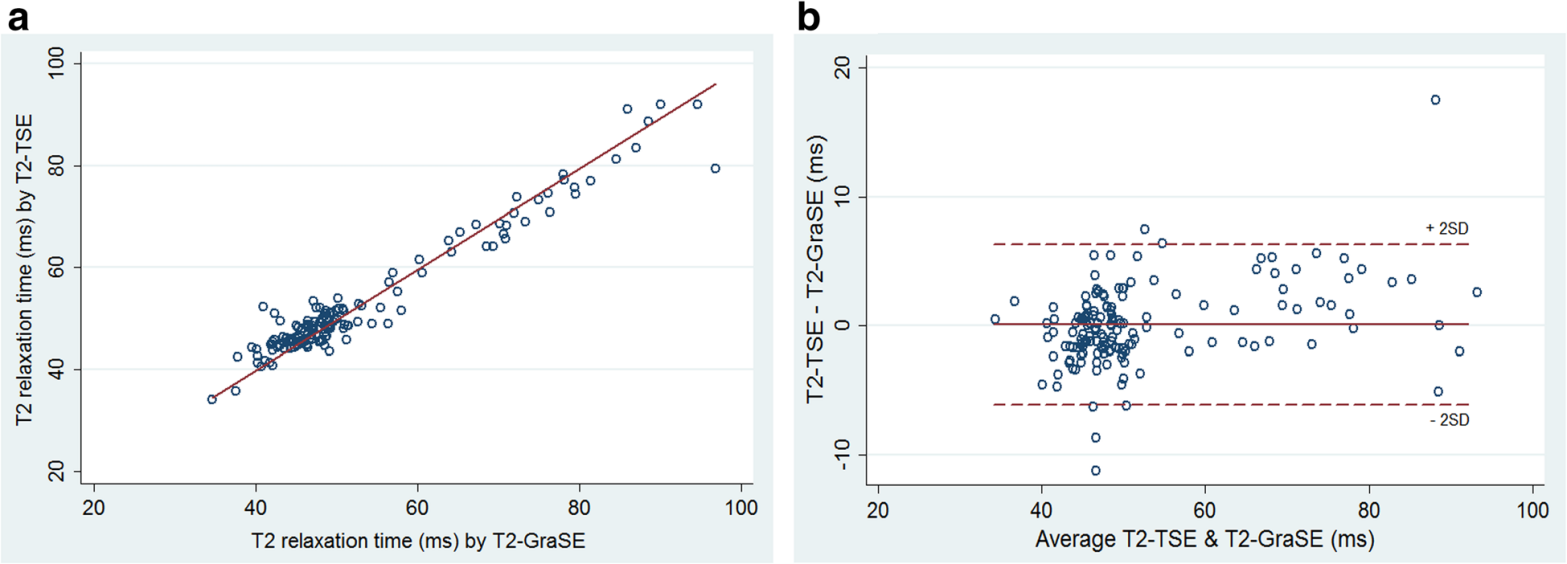
Assessment of agreement between T2 relaxation times measured by T2-TSE and T2-GraSE mapping. (**a**) Scatter plot showing almost perfect linear correlation (R^2^ = 0.99) of T2 relaxation times measured by T2-TSE and T2-GraSE mapping. (**b**) Bland-Altman analysis demonstrated excellent agreement between T2 relaxation readings from both T2-mapping sequences. The mean T2 relaxation time difference (T2-GraSE – T2-TSE) was 0.1 ms (agreement interval 95 %: −6.2 to 6.3 ms). The percentages of cases above and below the limits were 2.0 % and 2.7 %. The Spearman correlation coefficient between T2 relaxation time differences (T2-GraSE – T2-TSE) and mean T2 relaxation times (T2-GraSE & T2-TSE) was 0.33 (*p* < 0.001)

**Fig. 4:**
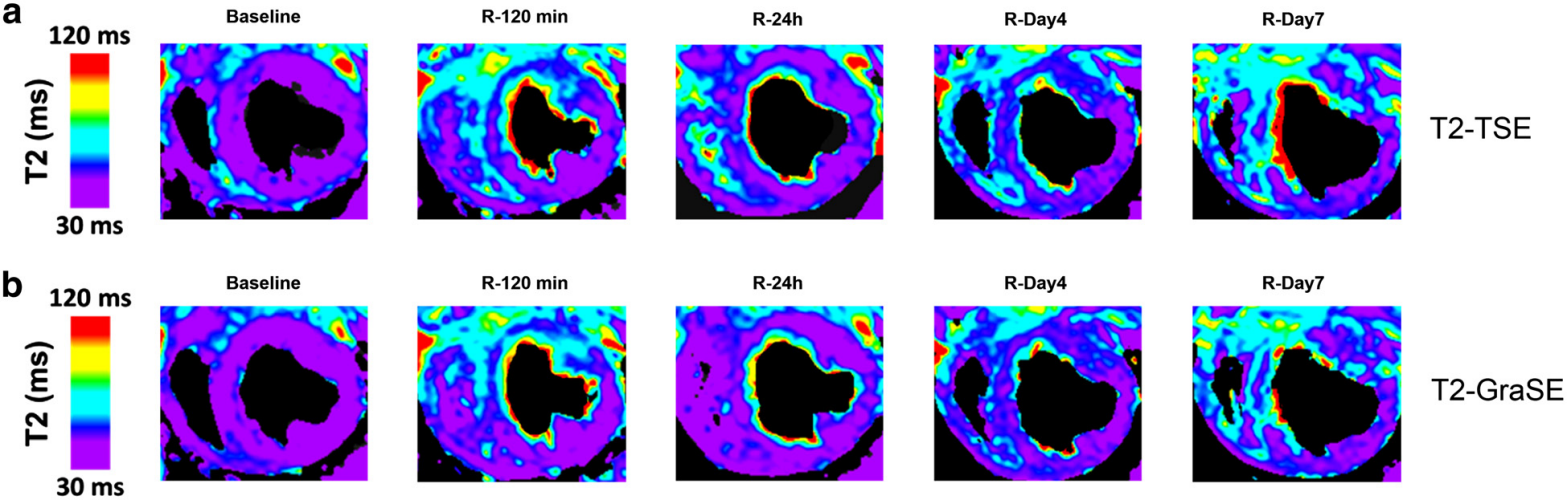
Representative images of serial T2-TSE and T2-GraSE mapping. Representative T2-mapping images for all time-points from the same pig subjected to 40 min I/R and sacrificed at day 7 after reperfusion. **a** T2-TSE images. **b** T2-GraSE images. All T2 maps were scaled between 30 and 120 ms. All sequences were acquired with black-blood preparation prepulse. For better visualization, generated T2 maps were masked to remove background signal. The masking was defined in the first echo image to improve the contrast between the cardiac muscle and the cavity. R: Reperfusion

T2 relaxation time measurements obtained by T2-TSE mapping and T2-GraSE mapping showed almost perfect correlation (R^2^ = 0.99, Fig. 3a). Intraclass correlation coefficients (ICC) evaluating absolute agreement and consistency of agreement between both T2 mapping techniques showed an excellent concordance between sequences (ICC > 0.96 for all evaluations, Table 3). Bland-Altman analysis showed a good agreement between sequences (Fig. 3b). Representative T2-mapping images obtained by T2-TSE and T2-GraSE from the same pig subjected to 40 min of ischemia and 7 days of reperfusion are shown in Fig. 4.

**Table 3:** Absolute agreement and consistency of agreement between T2 relaxation times measured by T2-TSE and T2-GraSE mapping

		ICC	95 % CI
Absolute agreement	Individual	0.967	0.954–0.976
	Average	0.982	0.976–0.988
Consistency of agreement	Individual	0.966	0.954–0.976
	Average	0.983	0.977–0.988

ICC: intraclass correlation coefficient. CI: confidence interval. TSE: turbo spin echo. GraSE: gradient spin echo

### Association between T2 relaxation time measurements and directly measured water content

**Fig. 5:**
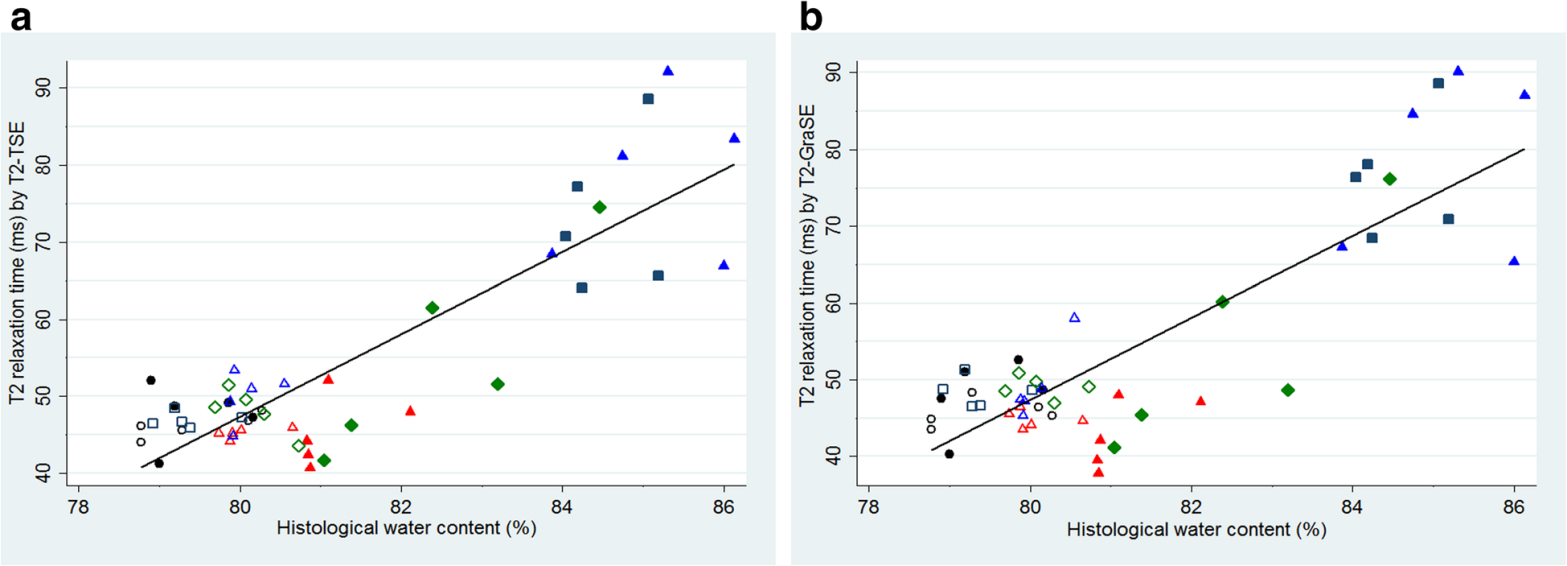
Association between T2 relaxation time and directly measured water content. **a** Scatter plot demonstrating good correlation between T2 relaxation times measured by T2-TSE mapping and directly measured myocardial water content. **b** Scatter plot demonstrating similarly good correlation between T2 relaxation times measured by T2-GraSE mapping and directly measured myocardial water content. For each panel, individual data represent values from pigs sacrificed at baseline (black circles), 120 min (navy squares), 24 h (red triangles), 4 days (green diamonds) and 7 days (blue triangles) after reperfusion. Solid symbols represent data for ischemic myocardium and hollow symbols represent data for remote myocardium. Therefore, a total of 50 individual points for each correlation are shown which corresponds to two samples per pig (ischemic and remote myocardium) from 5 groups of 5 pigs each sacrificed at the different time-points

Directly determined myocardial water content showed a good correlation with T2 relaxation time measurements performed by T2-TSE mapping (R^2^ = 0.75, *p* < 0.001; Fig. 5a) and by T2-GraSE mapping (R^2^ = 0.73, *p* < 0.001; Fig. 5b). No statistically significant differences between these correlations were observed.

## Discussion

In this study we present an in vivo validation of a CMR sequence for fast and accurate T2-mapping of the heart using the gradient-spin-echo (GraSE) technique that can be easily integrated in routine protocols, overcoming some of the limitations of current CMR mapping sequences for myocardial T2 quantification. For this endeavor we developed a closed-chest large animal model of ischemia/reperfusion in which animals had serial CMR scans and were sacrificed at serial time-points after reperfusion for direct quantification of myocardial water content. Two mapping sequences were used to quantify myocardial T2 relaxation time: the well-established reference T2-TSE technique and the newer T2-GraSE technique, which further speeds up the TSE sequence [18]. The translational pig model of myocardial infarction used in this study allows examination of a wide range of myocardial T2 relaxation times and myocardial water content [17], and produced values that closely mimic those clinically observed in several pathological conditions, strengthening the present validation.

Accurate noninvasive detection and quantification of myocardial edema is of great scientific and clinical interest given the occurrence of edema in several cardiovascular diseases and its usefulness for diagnosis and its correlation with ventricular remodeling and prognosis [20–22]. Many studies over the past decades have investigated the use of CMR with T2-weighted (T2W) sequences to monitor in the post-ischemic myocardium, since this approach is considered especially suited to the detection of high water content in this setting [23]. However, several problems inherent to T2W-CMR have limited the widespread uptake of this sequence for the detection of edema [6]. These problems include variations in surface coil sensitivity, motion artifacts, incomplete blood suppression, and the subjectivity of image interpretation [24].

A number of T2-mapping sequences have been recently proposed as a route to overcoming some of these limitations [7–9] and providing absolute quantification of regional T2 relaxation times that can be compared across studies. However, these methods are either time-consuming or require specialized software for data acquisition and/or post-processing, factors that impede their clinical routine use. Compared with these other approaches, T2-GraSE mapping has many advantages, including an acceptable acquisition time for integration into daily clinical CMR protocols, reduced energy requirements, and the use of standard post-processing methods. In this study, T2-GraSE mapping was 3-times faster than conventional reference standard T2-TSE mapping due to the interleaving of the EPI readout between two consecutive 180° pulses. The applicability of myocardial T2-mapping using the GraSE technique in humans has been reported recently [12, 25]; however, these studies mostly examined healthy hearts and therefore a narrow range of myocardial T2 relaxation times, and did not validate the sequence against directly determined tissue water content. Our study provides robust validation of T2-GraSE over a wide spectrum of myocardial T2 relaxation times and water contents that reflect a range of potential clinical scenarios.

Descriptions of previous T2-mapping sequences have relied on their ability to retrospectively identify the hypoperfused myocardial territory supplied by the occluded coronary artery—the area at risk—and there are no published data validating these techniques against true myocardial water content. Regional T2 relaxation time in the ischemic area can be altered depending on tissue characteristics [13, 14], the application of cardioprotective therapies [15, 16] or the timing of imaging acquisition [17], highlighting the need of establishing the relationship between T2-mapping and true myocardial water content directly quantified in the tissue. This question has been explored in only a few studies conducted over 20 years ago [26–30], and these studies were performed in low magnetic fields or with excised hearts, factors well known to affect T2 relaxation time [31]. The present study is thus the first to provide in vivo validation of T2-mapping against actual tissue myocardial water content in magnetic fields used in current clinical practice. We believe it is important to assess the association between actual water content and T2 relaxation time at 3 Tesla since no clear relationship has been established between these parameters at different field strength. In this regard, in vitro analysis have demonstrated an increase on myocardial T2 at 3 Tesla systems compared to 1.5Tesla [32], while in-vivo studies suggest equivalent T2 values at 3-Tesla with those previously reported at 1.5 Tesla [33].

Our data demonstrate similarly good correlation between myocardial water content and both T2-mapping techniques examined. In our study ≈ 25-30 % of T2 relaxation time variance was not completely explained by water content changes, highlighting the influence on T2 values of other tissue characteristics and components [6, 17] including the proportion of free/bound water as well as its location (intracellular/extracellular) in the tissue [34]. These data should be taken into account when interpreting clinical studies using these sequences.

In summary, we provide in vivo validation of a CMR sequence for fast and accurate T2-mapping of the heart using the gradient-spin-echo (GraSE) technique. This approach can be easily integrated in routine protocols since it is available for all equipment, and overcomes some of the limitations of current CMR mapping sequences for T2 quantification.

### Limitations

Although tissue changes in the post-I/R myocardium in pigs are similar to those in humans, we cannot rule out the existence of subtle histological differences between human and pig after infarction. As a consequence, the ≈ 30 % of T2 relaxation time variance that was not completely explained by water content changes in the present study might differ slightly from the value in humans. However, experimental studies allow validation, as shown here with the direct quantification of myocardial water content.

The pig is one of the most clinically translatable large animal models for the study of myocardial infarction and related issues due to its anatomical and functional similarities to humans [35], and also shows similar T2 values in the ischemic and remote myocardium to those seen in humans [8, 12].

In this study the ROIs for T2 relaxation time quantification covered the entire wall thickness and were individually adjusted by hand to carefully avoid the right and left ventricular cavities. The ROIs therefore might include different myocardial states (e.g., hemorrhage, microvascular obstruction, collagen) given that reperfused myocardium is a very heterogeneous condition [36]. However, we adopted this approach to match the analysis of water content, which was evaluated in the entire wall thickness. Both T2 mapping sequences did not apply any correction for respiratory motion. However, all scans were performed during free breathing; therefore animals had an abdominal breathing pattern with minimal chest movement in antero-posterior direction minimizing the ghosting artefacts in short axis view. Three-dimensional (3D) T2-mapping sequences have been very recently described and might benefit from higher spatial resolution, therefore reducing partial-volume averaging effects and misregistration between images [37, 38]. However, the proposed implementation of the T2-GraSE mapping can be performed in many modern scanners in a reasonable acquisition time, while 3D T2-mapping normally take longer acquisition times.

## Conclusions

We provide the first in vivo validation of T2-mapping for the assessment of myocardial edema. Given its shorter acquisition time, high accuracy in quantifying T2 relaxation time and no requirement for specific software for data acquisition or post-processing, fast T2-GraSE mapping of the heart is an attractive alternative to current CMR sequences for T2 quantification.

## Abbreviations

CMR: cardiovascular magnetic resonance
EPI: echo planar imaging
FOV: field of view
GraSE: gradient spin echo
I/R: ischemia/reperfusion
NEX: number of excitations
ROI: region of interest
T2W: T2 weighted
TE: echo time
TR: repetition time
TSE: turbo spin echo
